# Effects of the restrictive fluid strategy on postoperative pulmonary and renal function following pulmonary resection surgery

**DOI:** 10.1186/cc13334

**Published:** 2014-03-17

**Authors:** M Arslantas, H Batirel, B Bilgili, H Kara, B Yildizeli, M Yuksel, K Bostanci, A Kararmaz, I Cinel

**Affiliations:** 1 Marmara University Pendik Education and Research Hospital, Istanbul, Turkey; 2 Marmara University School of Medicine, Istanbul, Turkey

## Introduction

Goal-directed therapy used in the perioperative period of patients undergoing cardiac surgery shortens the length of ICU stay [1]. We aimed to compare the postoperative results of the liberal and restrictive fluid strategy used in patients undergoing pulmonary resection surgery (PRC).

## Methods

We have been using the restrictive fluid strategy since March 2013 in our institute. Patients who were on the liberal fluid regime were analyzed retrospectively. From March 2013 until today, patients who were on restrictive fluid strategy were analyzed prospectively. A total of 125 patients were included in the study. Age, duration of anesthesia, type of fluids given intraoperatively, fluid index (ml/kg/hour), fluid intake/output balance, creatinine and lactate levels were compared with pulmonary and renal morbidity, and length of stay in ICU, using multivariate analysis.

## Results

A significant correlation (*P *< 0.05) was established between the amount of crystalloid given intraoperatively, fluid index and fluid balance with pulmonary morbidity (*n *= 52). The fluid index and inotropes usage were correlated with the postoperative creatinine levels (*P *< 0.05). There was no correlation between perioperative lactate levels with fluid balance and fluid index. Intraoperative blood loss, the amount of given crystalloid, colloid, blood and FFP, fluid balance, duration of anesthesia and postoperative blood transfusion were found to be related (*P *< 0.05) with the length of ICU stay. Four percent of the patients required renal replacement therapy and the overall mortality was 0.8%.

## Conclusion

To reduce the morbidity of patients undergoing major surgery, the protocols of using the restrictive fluid strategy in the perioperative period and simultaneous protection of end organs, especially the kidneys, is currently the subject of this discussion. We observed that the restrictive fluid strategy did not lead to global organ hypoperfusion, which was monitored by lactate. Even though there was a negative correlation between the fluid index with creatinine levels and renal failure, the need for renal replacement therapy was observed only in one case. As a conclusion, the postoperative pulmonary morbidity and length of ICU stay can be reduced in patients who undergo PRC by using the restrictive fluid strategy (4.2 ± 0.3 ml/kg/hour), without causing any vital organ dysfunction.
